# The frequency and nature of prescribing problems by GPs-in-training (REVISiT): a retrospective review

**DOI:** 10.3399/BJGPO.2021.0231

**Published:** 2022-08-10

**Authors:** Nde-Eshimuni Salema, Brian G Bell, Kate Marsden, Gill Gookey, Glen Swanwick, Mindy Bassi, Rajnikant Mehta, Nick Silcock, Anthony J Avery, Richard Knox

**Affiliations:** 1 Division of Primary Care, School of Medicine, University Of Nottingham, Nottingham, UK; 2 NIHR Greater Manchester Patient Safety Translational Research Centre, University of Manchester, Division of Population Health, Health Services Research and Primary Care, Manchester, UK; 3 NHS Nottingham City PCT, Nottingham, UK; 4 Birmingham Acute Care Research/Institute of Applied Health Research (BCTU), Public Health Building, University of Birmingham, Edgbaston, UK; 5 NHS Nottingham City PCT, Wollaton Park Medical Practice, Nottingham, UK

**Keywords:** patient safety, family medicine, prescribing, prescriptions, general practice

## Abstract

**Background:**

Prescribing errors can cause significant morbidity and occur in about 5% of prescriptions in English general practices.

**Aim:**

To describe the frequency and nature of prescribing problems in a cohort of GPs-in-training to determine whether they need additional prescribing support.

**Design & setting:**

A primary care pharmacist undertook a retrospective review of prescriptions issued between 9 October 2014 and 11 March 2015 by 10 GPs in their final year of training from 10 practices in England.

**Method:**

Pre-existing standards and expert panel discussion were used to classify the appropriateness of prescribing. Data were imported into Stata (version 13) to perform descriptive analysis. An individualised report highlighting prescribing errors, suboptimal prescribing, and areas of good practice identified during the review was shared with the GPs-in-training and their trainers. This report was used to guide discussions during the GP-in-training’s feedback session.

**Results:**

A total of 1028 prescription items were reviewed from 643 consultations performed by 10 GPs-in-training. There were 92 prescribing errors (8.9%) and 360 episodes of suboptimal prescribing (35.0%). The most common types of error concerned medication dosages (*n* = 30, 32.6% of errors).

**Conclusion:**

Personalised review of prescribing revealed an error rate higher than recorded in a previous similar study mainly comprising GPs who had completed postgraduate training, and a substantially higher rate of suboptimal prescribing. A larger intervention study is now required to evaluate the effectiveness of receiving a personalised review of prescribing, and to assess its impact on patient safety.

## How this fits in

Prescribing is fundamental to the role of primary care clinicians and prescribing errors contribute to significant avoidable morbidity and mortality. GPs-in-training are a cohort of primary care clinicians who may benefit from additional support to enhance prescribing safety. Pharmacist-led review of the prescribing of individual GPs-in-training may facilitate personal reflection and practice change, as well as being able to highlight common errors, which can be used to enhance prescribing education for other GPs-in-training.

## Introduction

Prescribing errors can cause significant morbidity and mortality, which healthcare organisations are committed to reducing.^1,2^ The General Medical Council (GMC)-funded PRevalence And Causes of prescribing errors in general practiCe (PRACtICe) study showed that such errors occur in about 5% of all prescriptions in English general practices,^3^ with GPs-in-training identified as a group that may benefit from additional support to improve their prescribing. The PRACtICe study^3^ helped to identify several potential interventions that might reduce the prevalence of prescribing errors in general practice and the consequent risk of patient harm. GPs-in-training may lack a systematic and comprehensive education in safe prescribing^4,5^ and several studies have found that junior doctors benefit from additional training or support from a pharmacist.^4–7^ A promising translational application of the PRACtICe study^3^ was an educational intervention for GPs in training. This involved conducting a pharmacist-led review of prescribing to generate individualised feedback (REVISiT intervention). This article describes the pharmacist-led review, and reports the frequency and nature of prescribing problems detected. The qualitative findings will be reported elsewhere.

## Method

### Participants

Ten GPs-in-training were recruited from the East Midlands region of England in their final or penultimate year of training. The project was advertised through local GP training schemes. Consent from both GPs-in-training and their trainers was obtained before the reviews started.

### The REVISiT intervention

A primary care clinical pharmacist (GG) undertook a retrospective review of consultation notes on the practice clinical system (either SystmOne or EMIS Web) to identify where prescribing by a GP-in-training had taken place. GG had previously had her review work quality assured through her involvement with the PRACtICe study.^3^ Only prescriptions issued as a result of either a face-to-face or virtual (telephone) consultation were included in the review. Starting with the most recent consultation of the GP-in-training, the pharmacist worked backwards until approximately 100 consecutive prescription items had been identified. The consultations were collected between 9 October 2014 and 11 March 2015.

The pharmacist undertook a detailed review of the appropriateness of the medicines prescribed, along with a review of indication for the drug, dose, dosage instructions, quantities prescribed, and arrangements for medication review. As Table 1 outlines, the formulation of the drug was documented, as was its status within the consultation. Findings from the PRACtICe study guided the need to include these variables as part of the review.^3^

**Table 1.** Prescribing review definitions

Definition of prescribing error, suboptimal prescribing, and legal problem as outlined in the PRACtICe study.^3^	List of prescribing problem areas that errors and suboptimal prescribing can be attributed to	Drug classification by formulation type	Drug status — how was it prescribed within the consultation?
**Prescribing error:** ‘A clinically meaningful prescribing error occurs when, as a result of a prescribing decision or prescription writing process, there is an unintentional significant reduction in the probability of treatment being timely and effective or increase in the risk of harm when compared with generally accepted practice’. **Suboptimal prescribing:** these are prescribing problems that do not fit the above error definition, but represent less than ideal practice.**Legal problem:** these are prescribing problems that do not fit the above error definitions, but fall outside the legal framework for prescribing (an example would be prescribing for a whole family on a prescription for a single patient).	Unnecessary drug Incorrect drug Duplication Allergy error Contraindication error Interaction error Dose or strength error Formulation error Frequency error Timing error Information incomplete Generic or brand name error Omission error relating to failure to prescribe concomitant treatment Inadequate documentation in medical records Quantity error Inadequate review Duration error Monitoring not requested	Solid oral Topical Liquid oral Inhalers Eye or ear Vaginal Devices Injections Rectal	**New acute (NA):** a newly prescribed acute medication **Re-issued acute (RA):** a prescription of an acute medication that had previously prescribed for this patient by any prescriber **New Repeat (NR):** a prescription of a medication that was simultaneously added to the patient’s ‘repeat prescription’ **Amended Repeat (AR):** a prescription of one of the patient’s ‘repeat medications’ that had been amended during the consultation **Re-issued repeat (RR):** a prescription of one of the patient’s ‘repeat medications’ that had not been amended during the consultation

The definition of a prescribing error and suboptimal prescribing was the same as that used in the PRACtICe study.^3^ ‘Case law’ had been developed in the PRACtICe study, which facilitated the decision as to whether a prescribing scenario should be classified as a particular prescribing problem.^3^ Where potential prescribing problems did not fit within current case law, these were discussed at panel meetings involving a different pharmacist and two GPs from the team (TA, RK, and NES) to reach a consensus. The final agreed classification was entered on the database and the case law was updated. Extracts from the case law are available as Supplementary Table S1.

Prescriber, practice, and patient demographics (sex and age) were also collected, as these factors may influence prescribing safety.^3^ The weighted deprivation score, weighted by list size (http://fingertips.phe.org.uk/profile/general-practice/data), was calculated for the practices. These data were recorded on a Microsoft Access database. No patient-identifiable data were removed from the GP practices.

An individualised report was prepared for each GP-in-training. In keeping with good practice guidance in feedback, the report highlighted the prescribing problems identified, as well as examples of good practice observed.^8,9^ This report formed the basis for discussions held during a 1-hour tutorial with a clinical member of the research team (TA, RK, or GG), the GP-in-training, and their trainer. Participant interviews took place to assess the value of the intervention. The findings will be reported elsewhere.

### Statistical analysis

The pooled prevalence of all the prescribing problems identified across the 10 GPs-in-training were recorded. The PRACtICe study had also found it useful to report prescribing problems at the level of *British National Formulary* (BNF) chapter.^3^ Statistical analysis was performed with Stata (version 13) and SPSS (version 26). Categorical data were summarised with frequency counts and percentages, means and standard deviations (SD) were calculated for continuous variables (mean ± SD).

## Results

### Practice and participant characteristics

The characteristics of the 10 practices and GPs-in-training are described in Table 2. In terms of weighted deprivation score (weighted by list size), the average deprivation score for 2015 for the practices was 16.4 (SD = 9.5) There were equal numbers of male and female GPs-in-training who had their prescribing reviewed. Eight of the trainees had undertaken their training full-time, and two had done it part-time. Most of the trainees (90%) were in their final year of training. Further inferential statistical analysis based on participant characteristics was not performed owing to the small numbers in each category.

**Table 2.** Characteristics of practices and GPs involved in the review

**Practice characteristics**	**Practices, *n* (%)**
Type of practice	
Dispensing practice	8 (80)
Non-dispensing practice	2 (20)
Clinical system	
SystmOne	5 (50)
EMIS Web	5 (50)
Formulary availability on clinical system	
Formulary available	5 (50)
Formulary unavailable	5 (50)
Mean deprivation score based on IMD score^a^ (SD)	16.4 (9.5)
Mean list size (SD)	9392 (2499)
**GPs-in-training characteristics**	**Participants, *n* (%)**
		Sex of GP-in-training	
Male	5 (50)
Female	5 (50)
Ethnic group	
White British	5 (50)
British Indian	2 (20)
British Pakistani	1 (10)
Asian (other)	1 (10)
Mixed	1 (10)
Age range, years	
25–29	3 (30)
30–34	4 (40)
35–39	2 (20)
40–49	1 (10)
Date of graduation	
2004–2009^b^	5 (50)
2010	5 (50)
Country of graduation	
UK	8 (80)
Overseas	2 (20)
Stage of training	
ST2	1 (10)
ST3	9 (90)
Sex of trainer	
Male	7 (70)
Female	3 (30)

IMD = Index of Multiple Deprivation. SD = standard deviation. ST = specialty training. ^a^Deprivation score (IMD 2010; figures from 2012) http://fingertips.phe.org.uk/profile/general-practice/data. Higher IMD scores indicate greater relative deprivation.^b^One participant each for year 2004, 2005, 2007, 2008, and 2009.

### Consultations reviewed and prevalence of different types of prescription problems

The mean time GPs-in-training had been in their respective practices before one of their prescriptions was reviewed was 19.6 (SD = 7.0) weeks. It took an average of 1.8 (SD = 1.0) weeks’ worth of prescribing for a GP-in-training or 129.1 (SD = 32.4) consultations to be reviewed to achieve the desired 100 prescriptions for the pharmacist to review. A total of 1290 consultations conducted between 9 October 2014 and 11 March 2015 were reviewed by the pharmacist.

**Figure 1.**
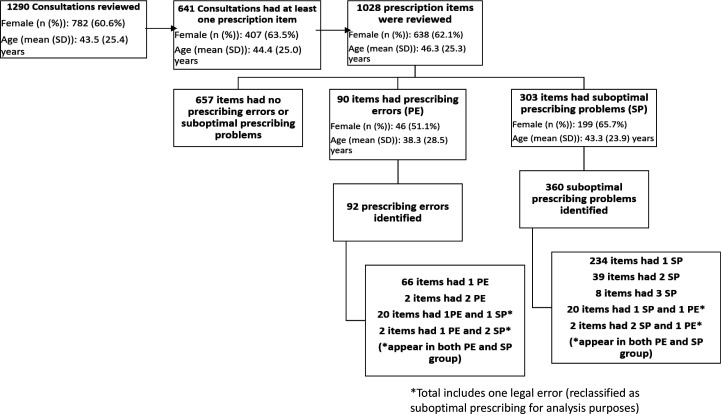
Flowchart showing number of consultations reviewed, prescription items reviewed, and a breakdown of prescribing problems

The number of prescription items reviewed was 1028 from the 641 consultations (Figure 1). All the GPs-in-training had at least one example of good prescribing highlighted, and examples are shown in Supplementary Table S2.

The breakdown of prescribing problems is shown in Figure 1. There were 452 prescribing problems: 92 prescribing errors (prevalence 8.9%), and 360 examples of suboptimal prescribing (35.0%).

Table 3 shows the prescribing from three *BNF* chapters (11, 12, and 15) was associated with a prescribing problem (errors and suboptimal prescribing) rate of ≥75%. External preparations (eye, ear, and topical) and oral preparations were commonly reported as being problematic. There was a large proportion of prescribing errors (35.3%) and many instances of suboptimal prescribing (30.9%) for liquid oral preparations. Most of the prescribing errors for the liquid orals (*n* = 16/24) were a result of incorrect antibiotic dosages being prescribed for children. More prescribing problems occurred for acute prescribing (new acute and reissued acute) than for repeat prescriptions. As seen in Table 3, most of the prescriptions with problems (73.0%) were for acute conditions, with the vast majority of errors and instances of suboptimal prescribing involving these prescriptions.

**Table 3.** Proportion of prescriptions reviewed with a prescribing problem by *British National Formulary* (BNF) chapter, formulation, and drug status

	**Items reviewed, *n* (%)**	**Proportion with an error, *n* (%)**	**Proportion with suboptimal prescribing, *n* (%)**
**Chapter of the *British National Formulary***			
Chapter 1: Gastro-intestinal system	112 (10.9)	5 (4.5)	36 (32.1)
Chapter 2: Cardiovascular system	121 (11.8)	2 (1.7)	15 (12.4)
Chapter 3: Respiratory system	65 (6.3)	6 (9.2)	22 (33.8)
Chapter 4: Central nervous system	201 (19.6)	27 (13.4)	53 (26.4)
Chapter 5: Infections	183 (17.8)	27 (14.8)	65 (35.5)
Chapter 6: Endocrine system	46 (4.5)	3 (6.5)	25 (54.3)
Chapter 7: Obstetrics, gynaecology, and urinary-tract disorders	39 (3.8)	0 (0)	11 (28.2)
Chapter 8: Malignant disease and immunosuppression	1 (0.1)	0 (0)	0 (0)
Chapter 9: Nutrition and blood	9 (0.9)	1 (11.1)	4 (44.4)
Chapter 10: Musculoskeletal and joint diseases	66 (6.4)	4 (6.1)	22 (33.3)
Chapter 11: Eye	16 (1.6)	3 (18.8)	13 (81.3)
Chapter 12: Ear, nose, and oropharynx	40 (3.9)	2 (5.0)	30 (75.0)
Chapter 13: Skin	125 (12.2)	12 (9.6)	61 (48.8)
Chapter 14: Immunological products and vaccines	1 (0.1)	0 (0)	0 (0)
Chapter 15: Anaesthesia	3 (0.3)	0 (0)	3 (100.0)
**Total**	**1028** (**100.0**)	**92** (**8.9**)	**360** (**35.0**)
	**Items reviewed, *n* (%)**	**Proportion with an error, *n* (%)**	**Proportion with suboptimal prescribing, *n* (%)**
**Formulation type**			
Solid oral	681 (66.2)	44 (6.5)	195 (28.6)
Topical	173 (16.8)	14 (8.1)	92 (53.2)
Liquid oral	68 (6.6)	24 (35.3)	21 (30.9)
Inhalers	48 (4.7)	6 (12.5)	20 (41.7)
Eye or ear	30 (2.9)	4 (13.3)	25 (83.3)
Vaginal	12 (1.2)	0 (0)	6 (50.0)
Devices	11 (1.1)	0 (0)	0 (0)
Injections	3 (0.3)	0 (0)	0 (0)
Rectal	2 (0.2)	0 (0)	1 (50.0)
**Total**	**1028** (**100.0**)	**92** (**8.9**)	**360** (**35.0**)
**Drug status**			
New acute (NA)	666 (64.8)	65 (9.8)	271 (40.7)
Reissued acute (RA)	84 (8.2)	10 (11.9)	34 (40.5)
New repeat (NR)	65 (6.3)	1 (1.5)	17 (26.2)
Amended repeat (AR)	3 (0.3)	0 (0)	1 (33.3)
Reissued repeat (RR)	210 (20.4)	16 (7.6)	37 (17.6)
**Total**	**1028** (**100.0**)	**92** (**8.9**)	**360** (**35.0**)

The proportions of prescribing errors and suboptimal prescribing identified are presented in Table 4 by classification type. The two most common types of error reported for the GPs-in-training were dose or strength error (32.6%) and incomplete information (26.1%).

**Table 4.** Distribution of prescribing events by classification type as determined by case law

Prescribing problem	Items with an error, *n* (%)	Items with suboptimal prescribing, *n* (%)
Unnecessary drug	5 (5.4)	6 (1.7)
Incorrect drug	7 (7.6)	30 (8.3)
Duplication	5 (5.4)	13 (3.6)
Allergy error	1 (1.1)	0 (0)
Contraindication error	3 (3.3)	0 (0)
Interaction error	1 (1.1)	0 (0)
Dose or strength error	30 (32.6)	39 (10.8)
Formulation error	2 (2.2)	15 (4.2)
Frequency error	1 (1.1)	2 (0.6)
Timing error	0 (0)	15 (4.2)
Information incomplete	24 (26.1)	117 (32.5)
Generic or brand name error	0 (0)	8 (2.2)
Omission error relating to failure to prescribe concomitant treatment	5 (5.4)	7 (1.9)
Inadequate documentation in medical records	3 (3.3)	52 (14.4)
Quantity error	3 (3.3)	23 (6.4)
Inadequate review	2 (2.2)	8 (2.2)
Duration error	0 (0)	24 (6.7)
Monitoring not requested	0 (0)	1 (0.3)
**Total**	**92** (**100.0**)	**360** (**100.0**)

Supplementary Table S3 provides examples of prescribing problems identified during the review and the recommendation to the GP-in-training suggested by the pharmacist.

## Discussion

### Summary

Ten GPs-in-training each had approximately 100 sequential prescription items retrospectively reviewed by a primary care clinical pharmacist. The consultations covered an average period of 2 weeks of prescribing. A total of 1028 items were reviewed, which revealed 452 prescribing problems. There were 92 prescribing errors (prevalence: 8.9% of items prescribed) and 360 episodes of suboptimal prescribing (prevalence: 35.0%). The two most common types of error reported were dose or strength error (32.6%) and incomplete information (26.1%).

### Strengths and limitations

Previous studies have suggested that GPs-in-training may have additional educational needs with respect to prescribing,^3,5^ with effective feedback characterised as being free of blame and non-judgemental.^10^ This is the first study of its kind to systematically scrutinise the quality of prescribing by GPs-in-training in the UK. Consistency of classification of prescribing problems was maintained by using pre-existing case law, which was developed through the PRACtICe study.^3^ Where a prescribing problem could not be categorised using case law, this was decided by panel discussion. As this was a pilot study, only one pharmacist was utilised. Their review work had previously been validated through the PRACtICe study.

This study involved only 10 GPs-in-training and so the results cannot be reliably extrapolated to the entire population of GPs-in-training in the UK. The GPs-in-training were all located in one region (East Midlands, England), which is a further limitation to the generalisability of the findings. The average deprivation score for 2015 for the practices was 16.4 (SD = 9.5) while the English average was 21.8, which means that the practices were slightly less deprived. However, the percentage of female consultations (61%) was similar to the consultation rate reported (2013) for the UK.^11^

### Comparison with existing literature

The PRACtICe study is the most comprehensive review of prescribing in English primary care. It reported that errors occurred in about 5% of prescriptions, and that suboptimal prescribing occurred in 7% of items. Serious errors occurred at a rate of one in 550 items. The PRACtICe study highlighted GPs in training as a cohort of prescribers in need of additional support.^3^ One of the translational applications of the PRACtICe^3^ study was an educational intervention that involved conducting a pharmacist-led review of prescribing involving individualised feedback (REVISiT intervention).

Other studies have reported an increase in prescribing errors among doctors-in-training. The EQUIP (Enhancing the quality of user involved care planning in mental health services) study reviewed a total of 124,260 prescription orders across 19 hospitals. The error rate for prescriptions issued by consultants was reported at 5.9%, whereas that of foundation year 2 doctors was 10.9%, and specialty training doctors 8.3%.^12^ These figures are comparable with the 8.9% error rate reported in the present study of GPs-in-training. A study in the US reviewed more than 2000 prescriptions issued by doctors in various training programmes. The error rate reported for those in a family medicine training programme was 11%.^13^

The proportion of items that were prescribed suboptimally was markedly higher in the present study than the rate reported in the PRACtICe study (34.9% versus 7%).^3^ In their review article, Hanlon *et al* commented that the ‘definitions for suboptimal prescribing are numerous, and measurement varies from study to study’.^14^ This therefore makes rate comparisons difficult. However, the same definition for both studies was used, although the authors are aware that the pharmacist in the current study (GG) was looking particularly carefully for suboptimal prescribing in order to ensure maximum educational benefit when prescribing problems were fed back to the GPs-in-training, which may have led to a risk of bias. Nevertheless, with such large differences between the two studies, it is likely that the trainees had not fully learnt the skills of high quality prescription writing. The sample size of the present study should also be considered here.

The most common types of error identified were dose or strength error (32.6%) and incomplete information (26.1%). These categories were similarly highly represented in the PRACtICe study (17.8% and 30.0%, respectively),^3^ and also in a study involving 55 care homes across the UK (14.4% and 37.9%, respectively).^15^ It is likely that GPs-in-training would benefit from education on how to avoid these errors.

There was a large proportion of prescribing errors and many instances of suboptimal prescribing for liquid oral preparations. Liquid oral preparations may be prone to medication errors because they often require the calculation of patient-specific doses.^16,17^ Furthermore, in the present study, many of the errors from the ‘liquid oral’ category related to paediatric antimicrobial prescribing. Other authors have reported that antibiotic prescribing is a particularly challenging area for junior doctors.^18,19^ The findings are especially relevant in the current age of increasing antibiotic stewardship requirements.^20–22^

Most of the prescriptions (73.0%) were for acute conditions, with the vast majority of errors and instances of suboptimal prescribing involving these prescriptions. A study that compared the workload of trainee GPs and their trainers found that trainees tended to see more acute cases and fewer patients with chronic conditions,^23^ although it should be noted that there was an increase in acute cases for GPs during the winter months.^24^ The present findings may reflect that patients with chronic conditions are more likely to choose to consult with a GP that they have a longer-term relationship with, which is more likely to be a more experienced GP.^25^ However, to fully prepare GPs-in-training for their qualified role, it is important that they are given opportunities to gain the necessary experience of managing patients with chronic conditions. The results of a systematic review looking into training for postgraduate doctors has indicated that not much is known about the availability and impact of education and training for postgraduate medical doctors in the area of dealing with patients with multiple morbidities.^26^

### Implications for research and practice

GPs-in-training are a group of prescribers who may benefit from additional support. Personalised review of prescribing revealed an error rate higher than that from a previous similar study mainly comprising GPs who had completed postgraduate training, and a high rate of suboptimal prescribing. Having an awareness of these problems may help GPs-in-training plan their learning and assist those training them. Trends in the data demonstrate that particular types of error that continue to be highlighted, such as those relating to dose or strength, and those involving incomplete information on prescriptions, should also be used to influence prescribing education more widely. Findings from the REVISiT intervention have already been used to inform guidance given to GPs-in-training in the UK who are undertaking their prescribing assessment.^27^ This assessment is based on principles of self-review and is now mandatory for doctors in their final year of GP training.^27^

The increasing burden of chronic disease is well documented; for example, at least 50% of GP appointments in the UK are made by patients with chronic conditions.^28^ The high proportion of acute prescribing revealed in the present study could suggest that trainees may benefit from wider exposure to chronic cases to better prepare them for future prescribing practice.

This study showed that within a 2-week period, GPs-in-training did sufficient prescribing to obtain a sample of 100 prescriptions. This finding is important when considering the investment of resources for conducting an intervention, such as REVISiT, in everyday general practice. The majority of the GPs in this study were in their final year of training, some of the prescriptions reviewed were issued as late as 5 months before training was completed. It is possible that additional educational input regarding prescribing would continue to be beneficial even beyond specialist training. Educational activities, such as e-learning, are able to provide generic guidance, and have proven utility in the postgraduate domain.^29–31^ The additional benefit of targeted, individualised input should be explored. Further research in this area would be prudent. A larger intervention study is now required to evaluate the effectiveness of receiving a pharmacist-led personalised review of prescribing, and to fully assess its impact on patient safety.

## Data

The dataset relied on in this article is available from the corresponding author on reasonable request.
